# Virtual high-throughput screening: Potential inhibitors targeting aminopeptidase N (CD13) and PIKfyve for SARS-CoV-2

**DOI:** 10.1515/biol-2022-0637

**Published:** 2023-07-07

**Authors:** Zijing Ruan, Jiaxi Tang, Mingtang Zeng, Ping Fan

**Affiliations:** Department of Clinical Pharmacy, West China Hospital, Sichuan University, Chengdu, Sichuan, 610041, China

**Keywords:** SARS-CoV-2, virtual screening, potential inhibitor, CD13, PIKfyve, drug repurposing

## Abstract

Since the outbreak of the novel coronavirus nearly 3 years ago, the world’s public health has been under constant threat. At the same time, people’s travel and social interaction have also been greatly affected. The study focused on the potential host targets of SARS-CoV-2, CD13, and PIKfyve, which may be involved in viral infection and the viral/cell membrane fusion stage of SARS-CoV-2 in humans. In this study, electronic virtual high-throughput screening for CD13 and PIKfyve was conducted using Food and Drug Administration-approved compounds in ZINC database. The results showed that dihydroergotamine, Saquinavir, Olysio, Raltegravir, and Ecteinascidin had inhibitory effects on CD13. Dihydroergotamine, Sitagliptin, Olysio, Grazoprevir, and Saquinavir could inhibit PIKfyve. After 50 ns of molecular dynamics simulation, seven compounds showed stability at the active site of the target protein. Hydrogen bonds and van der Waals forces were formed with target proteins. At the same time, the seven compounds showed good binding free energy after binding to the target proteins, providing potential drug candidates for the treatment and prevention of SARS-CoV-2 and SARS-CoV-2 variants.

## Graphical abstract

**Figure:**
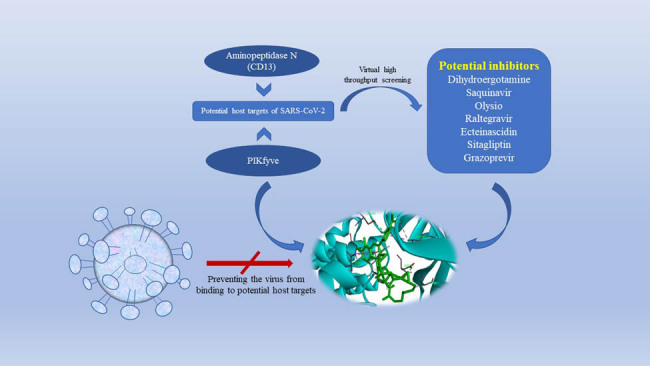

## 1 Introduction

COVID-19, caused by the severe acute respiratory syndrome coronavirus 2 (SARS-CoV-2), has been circulating worldwide since 2019 [1]. According to statistics from the World Health Organization, as of May 5, 2023, the number of global deaths caused by this epidemic has reached 6,800,000, involving 35 countries. SARS-CoV-2 bel to the genus Betacoronavirus, which is an enveloped, positive-sense, single-stranded RNA virus with 26–32 kb genome [2,3]. In addition, the genus includes the severe acute respiratory syndrome coronavirus (SARS-CoV), which was endemic in 2002–2003, Middle East Respiratory syndrome coronavirus (MERS-CoV), human coronaviruses (HCoV)-OC43, and HCoV-HKU1 [4]. Among them, SARS-CoV-2 shares 80% identity with SARS-CoV and 50% similarity with MERS-CoV, which caused global outbreaks in 2011 [5]. The outbreak of these coronaviruses, especially SARS-CoV-2, has caused serious impacts on human health around the world, and at the same time, it has also caused huge losses to the global economy. So far, more than 30 vaccines worldwide have been approved for marketing or urgently authorized for use. At the same time, there are nearly 30 drugs globally approved for COVID-19, including neutralizing antibodies, monoclonal antibodies, neuropeptide hormones, and small-molecule drugs. Among them, the approved small-molecule antiviral drugs are represented by Remdesivir, Paxlovid, Molnupiravir, and Azvudine. Although vaccines and drugs against SARS-CoV-2 have been developed to prevent the outbreak of the virus pandemic, due to the high mutagenicity of the novel coronavirus genome, such as the rapidly spreading Omicron variant, the existing strategies cannot fully guarantee the subsequent effectiveness [6]. Therefore, we urgently need to find new targets and develop small-molecule antiviral drugs for the huge threat posed by COVID-19. The development of small-molecule drugs is also the reuse or repositioning of drugs, with low cost and high accessibility.

Aminopeptidase N (CD13), an extracellular enzyme, is also a zinc-dependent metalloproteinase whose N-terminus is anchored to the cell membrane and faces the extracellular catalytic domain [7]. Expression of this cell-surface glycoprotein is found in many human tissues, including the lung [8]. In addition, the protease is involved in peptide cleavage, viral infection, endocytosis, and cell signal transduction [9]. Studies have shown that CD13 is not only a receptor for human coronavirus 229E, but also a receptor for gut-borne coronavirus, which also indicates that CD13 may also be involved in COVID-19 invasion [10,11]. Therefore, as one of the host receptors of coronavirus, CD13 can also be considered a potential target for SARS-CoV-2 to deal with the possible emergence of coronavirus variants in the future.

PIKfyve, a phosphoinositol 5-kinase, synthesizes PtdIns5P and PtdIns(3,5) diphosphate, which in turn regulates membrane homeostasis [6]. PIKfyve plays a key role in endocytosis of viral entry into host cells [12]. Studies have shown that PIKfyve inhibitors may prevent the viral/cell membrane fusion stage, resulting in the failure of viral single-stranded RNA release into the cytoplasm, thereby terminating SARS-CoV-2 entry into host cells. In addition to this, the rapid generation of vacuoles and inactivated tissue proteins induced by PIKfyve inhibitors also severely disrupted the new life cycle of the virus, thereby reducing infection [6]. Therefore, drug targeting PIKfyve to interfere with the endocytosis of host cells is an effective way to block virus infection [13]. It is a good idea to use PIKfyve as a potential target against SARS-CoV-2.

Therefore, in this study, CD13 and PIKfyve were used as two target proteins for drug screening, to find potential drugs against SARS-CoV-2. A computer virtual screen in a database of known drugs can help rapidly identify potential drug candidates for COVID-19 prevention and treatment. More importantly, previous literature has shown that some of the virtually screened compounds, such as ribavirin and ritonavir, have been shown to be effective in the treatment of COVID-19 [14]. It takes approximately a decade of research to develop new drugs. Therefore, reusing small-molecule drugs may be an effective strategy to deal with SARS-CoV-2 infection [15]. Through high-throughput screening methods, we hope to find potential antiviral drug candidates.

## 2 Materials and methods

### 2.1 Construction of small molecular ligands and preparation of target proteins

The Food and Drug Administration (FDA) compound sublibrary was downloaded from the zinc database (http://zinc.docking.org/substances/subsets/). Then, the model was converted to pdbqt format by prepare_receptor4.py script by assigning atomic types and atomic charges. All rotatable bonds in the molecule are set to be flexible for flexible docking. The crystal structure of CD13 (PDB ID: 4FYT) was used as the target protein of SARS‐CoV‐2. The crystal structure of PIKfyve (PDB ID: 7K1W) was used as the target protein of SARS‐CoV-2.

### 2.2 Molecular docking

First, the 3D structure of the FDA-approved compound was saved after energy minimization, and the pdbqt format was generated by AutoDock Tools software. Log in to the PDB database to download the 3D crystal structure of the target protein and recalculate the charge after dewatering, ligand, and hydrogenation. Then, the compound structure file is imported to detect the total charge, assign the charge, and check the flexible rotatable. Finally, AutoDock Vina was used for molecular docking and the binding energy was calculated. The absolute value of binding energy >4.25 indicates that the molecule has initial binding ability with the target, >5.0 indicates strong binding ability, and >7.0 indicates strong binding ability.

### 2.3 Molecular dynamics (MD) simulation and binding free energy calculation

The Gromacs 2020 software package was used to simulate the MD of the screened receptor protein–small molecule complex. AMBER99SB-ILDN field parameters were used for proteins and gaff general field parameters were used for small-molecule ligands. The sobtop program was used to construct the small-molecule topology and RESP was used for charge fitting. The TIP3P dominant water model was selected, and the minimum distance between the atoms in the protein and the edge of the water box was 1.0 nm. Sodium or chloride ions were used to neutralize the system charge depending on the docking result. The work flow of MD simulation includes four steps: energy minimization, heating, balancing, and production dynamics simulation. Target equilibrium temperature is 300 K. Finally, MD simulations were performed for 50 ns, calculating and recording conformations every 2 ps. In the simulation process, the simulated unframe is isotropic and periodic boundary conditions are applied. After the MD simulation, we calculated the root mean square deviation (RMSD), root mean square fluctuation (RMSF), radius of gyration (*R*_g_), and solvent accessible surface area (SASA) of the protein. Finally, the hydrogen bond interaction between the target protein and the compound was calculated. The g_MMPBSA method using the Gromacs 2020 program was used to calculate the binding free energy of ligands and proteins.

## 3 Results and discussion

### 3.1 Docking results of 2631 drugs on CD13 and PIKfyve, potential targets of SARS-CoV-2

**Figure 1:**
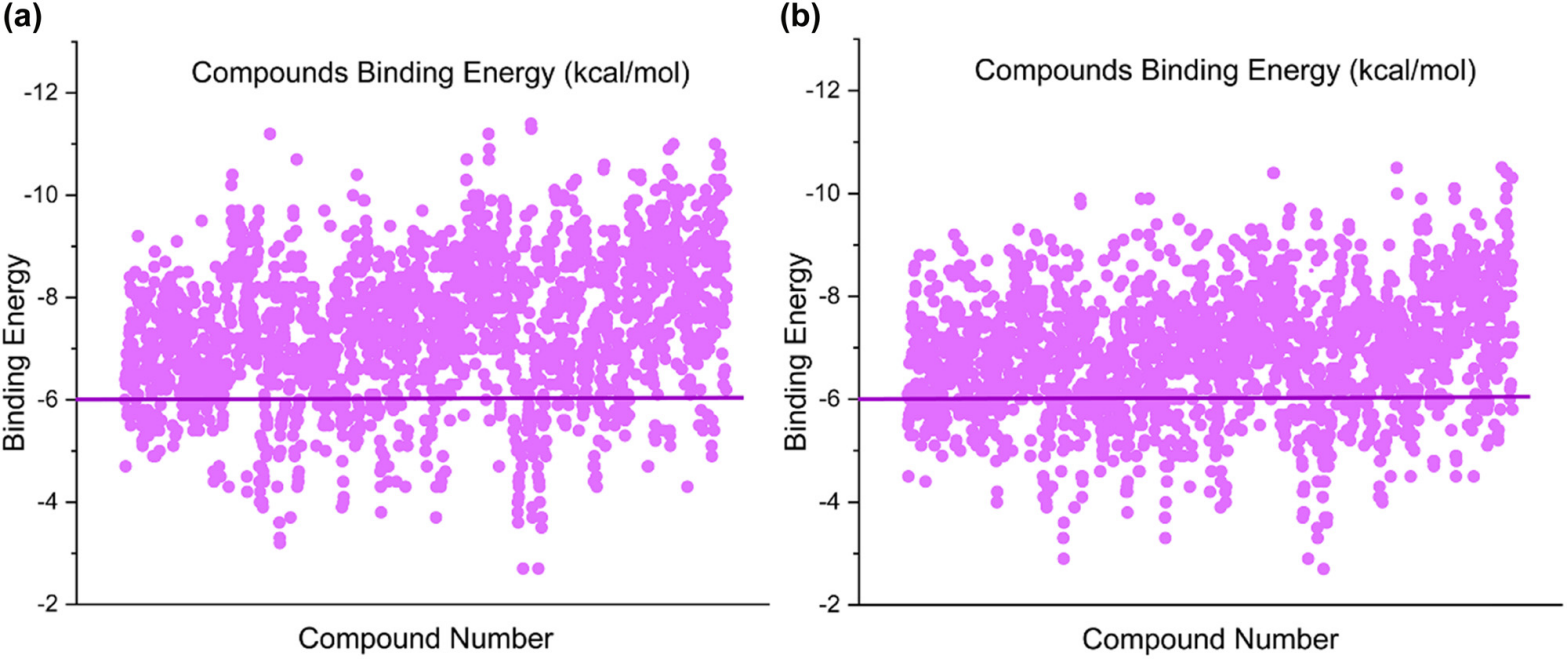
Binding energies for virtual hit compounds. The violet line shows the cut-off range for the compounds selected. (a) Binding energy of CD13 with FDA-approved compounds. (b) Binding energy of PIKfyve with FDA-approved compounds.

A total of 2,631 FDA-approved small molecules were screened for molecular docking. The AutoDock tool was used to calculate the binding energy of the compound. The compound selected according to the determined cutoff value −6.00 kcal/mol is shown in Figure 1. The purple line shows the cutoff range. The results showed that 1,801 FDA-approved compounds showed binding energies higher than −6.00 kcal/mol with potential target CD13. Meanwhile, 1666 FDA-approved compounds showed binding energies higher than −6.00 kcal/mol with potential targets PIKfyve. According to the results of molecular docking between APN and compounds, the 10 top compounds with affinities between −10.1 and −11.4 kcal/mol were selected (Table 1). In the same way, the 10 top compounds with affinities between −9.5 and −10.4 kcal/mol were selected from the results of molecular docking between PIKfyve and the compounds (Table 2). According to the selected compounds, dihydroergotamine, Saquinavir, and Olysio showed high affinity for both target proteins CD13 and PIKfyve with good docking scores. In addition, Saquinavir, Olysio, Raltegravir, and Grazoprevir were all strongly associated with antiviral activity.

**Table 1:** Ten compounds screened with CD13 as the target protein

Compounds name	ID	Data	Affinity (kcal/mol)
Nilotinib	ZINC000006716957	FDA	−11.4
Dihydroergotamine	ZINC000003978005	FDA	−11.2
Lumacaftor	ZINC000064033452	FDA	−11
Ergotamine	ZINC000052955754	FDA	−10.9
Saquinavir	ZINC000003914596	FDA	−10.7
Olysio	ZINC000164760756	FDA	−10.6
Raltegravir	ZINC000013831130	FDA	−10.2
Ecteinascidin	ZINC000150338708	FDA	−10.2
Grazoprevir	ZINC000095551509	FDA	−10.1
Tipranavir	ZINC000100016058	FDA	−10.1

**Table 2:** Ten compounds screened with PIKfyve as the target protein

Compounds name	ID	Data	Affinity (kcal/mol)
Dihydroergotamine	ZINC000003978005	FDA	−10.4
Ergotamine	ZINC000052955754	FDA	−10.1
Sitagliptin	ZINC000001489478	FDA	−9.9
Olysio	ZINC000164760756	FDA	−9.9
Nilotinib	ZINC000006716957	FDA	−9.6
Grazoprevir	ZINC000095551509	FDA	−9.6
Saquinavir	ZINC000029416466	FDA	−9.5
Ecteinascidin	ZINC000150338708	FDA	−9.4
Haloperidol	ZINC000000537822	FDA	−9.3
Naldemedine	ZINC000100378061	FDA	−9.5

### 3.2 Docking results of dihydroergotamine against CD13 and PIKfyve

**Figure 2:**
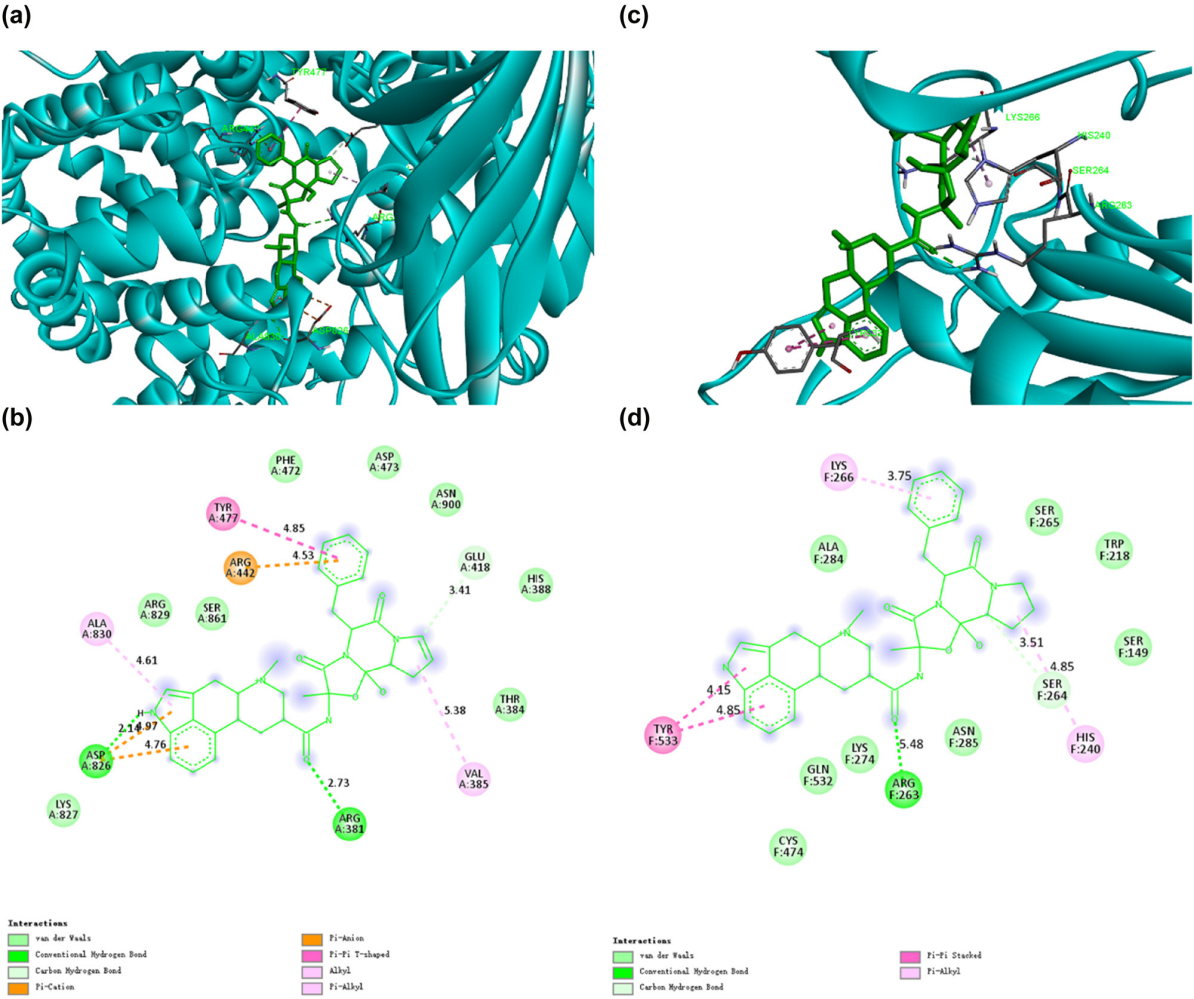
The binding model of dihydroergotamine against CD13 and PIKfyve. (a) Interactions between dihydroergotamine (green) and associated residues (purple) in the CD13 protein interface pocket (cyan) for SARS‐CoV‐2. (b) Two-dimensional interaction diagram of dihydroergotamine binding to the CD13 active site. (c) Interactions between dihydroergotamine (green) and associated residues (purple) in the PIKfyve protein interface pocket (cyan) for SARS‐CoV‐2. (d) Two-dimensional interaction diagram of dihydroergotamine binding to the PIKfyve active site. The numbers next to the dotted line indicate the interaction distance.

Dihydroergotamine (DHE), as an alpha-adrenergic antagonist, is structurally similar to many neurotransmitters, such as dopamine and epinephrine, so it can bind to many receptors. Until now, DHE is still the drug of choice for cluster headache, migraine state, and acute migraine [16]. Recent studies have shown that DHE also plays a certain role in potential antiviral [17,18]. Our docking study showed that DHE had high binding capacity with CD13 and PIKfyve, potential targets of SARS-CoV-2, with binding energies −11.2 and −10.4 kcal/mol, respectively. According to Figure 2a and b, when DHE binds to CD13, two conventional hydrogen bonds are formed, involving ASP862 and ARG381. One carbon–hydrogen bond is formed with GLU418. It also formed eight van der Waals, which involve LYS827, ARG829, SER861, PHE472, ASP473, ASN900, HIS388, and THR384. In addition, DHE made one cation and anion Pi interaction with ARG442 and one Pi–Pi T-shaped interaction with TYR477. Besides, DHE had two Pi-alkyl with ALA830 and VAL385. According to Figure 2c and d, when DHE binds to PIKfyve, eight van der Waals are formed with CYS474, GLN532, LYS274, ASN285, SER149, TRP218, SER265, and ALA284. Besides, DHE had one conventional hydrogen bond with ARG263 and one carbon–hydrogen bond with SER264. In addition, DHE made two Pi-alkyl with LYS266 and HIS240. It also had one Pi–Pi stacked with TYR533. Previously, studies have shown that DHE has strong binding ability with SARS-CoV-2 3C-like protease (3CLpro) and viral RNA-dependent RNA polymerase (RdRp), thereby inhibiting the activities of these two enzymes [19]. Our screening study further found that DHE can bind to CD13 and PIKfyve and, thus, may block the interaction of SARS-CoV-2 with potential human targets.

### 3.3 Docking results of Saquinavir against CD13 and PIKfyve

**Figure 3:**
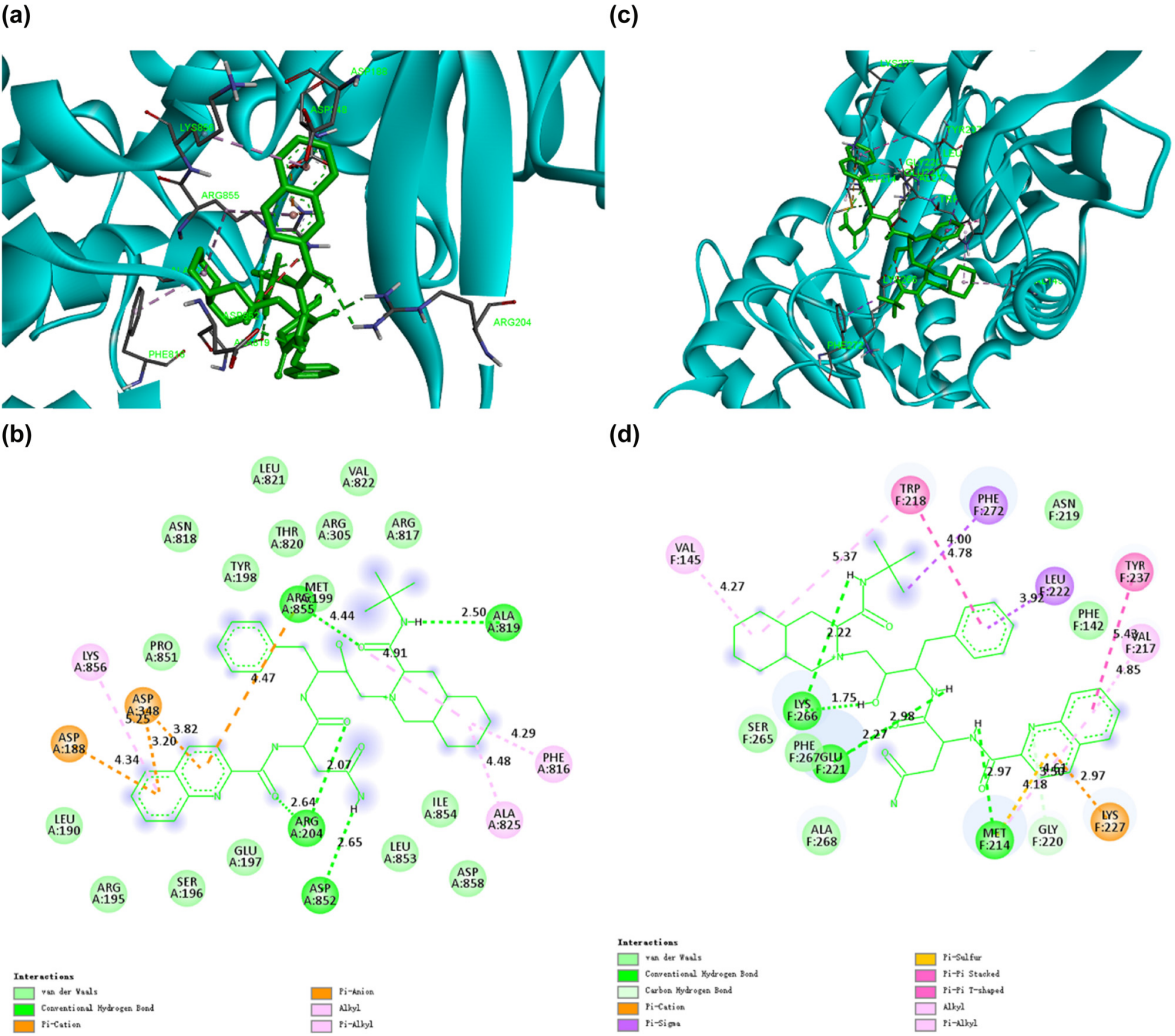
The binding model of Saquinavir against CD13 and PIKfyve. (a) Interactions between Saquinavir (green) and associated residues (purple) in the CD13 protein interface pocket (cyan) for SARS‐CoV‐2. (b) Two-dimensional interaction diagram of dihydroergotamine binding to the CD13 active site. (c) Interactions between Saquinavir (green) and associated residues (purple) in the PIKfyve protein interface pocket (cyan) for SARS‐CoV‐2. (d) Two-dimensional interaction diagram of dihydroergotamine binding to the PIKfyve active site. The numbers next to the dotted line indicate the interaction distance.

Saquinavir, first approved by the US FDA in 1995, was the first PI to be used to treat HIV. Saquinavir is a protease inhibitor that can bind strongly with protease, resulting in competitive inhibition of its activities required for maturation and proliferation, thus forming an immature provirus that cannot infect cells [20]. Many studies have shown that Saquinavir plays an important role in antiviral treatment [21,22]. Our docking results showed that Saquinavir could bind closely to CD13 and PIKfyve, potential targets of SARS-CoV-2, with binding energies of −10.7 and −9.5 kcal/mol, respectively. As shown in Figure 3a and b, when Saquinavir binds to CD13, it forms 16 van der Waals forces with amino acid residues LEU190, ARG195, SER196, GLU197, LEU853, ILE854, ASP858, PRO851, ASN818, TYR198, MET199, THR820, LEU821, ARG305, ARG817, and VAL822, respectively. Meanwhile, four conventional hydrogen bonds were formed with residues ARG204, ASP852, ARG855, and ALA819, respectively. It also made two cation and anion Pi interactions with ASP188 and ASP348. Besides, Saquinavir had three Pi-alkyl with LYS856, ALA825, and PHE816. According to Figure 3c and d, when Saquinavir binds to PIKfyve, it forms five van der Waals interactions with SER265, PHE267, ALA268, ASN219, and PHE142. It also forms three conventional hydrogen bonds and one carbon–hydrogen bond with LYS266, GLU221, MET214, and GLY220. Moreover, two Pi-sigma are formed between Saquinavir and PIKfyve, involving PHE272 and LEU222. In addition, Pi–Pi stacked and Pi–Pi t-shaped are formed with TRP218 and TYR237. Meanwhile, Pi-alkyl is formed between Saquinavir and the amino acid residue VAL217 of PIKfyve. Chiou et al. showed that Saquinavir blocks the proteolytic activity of SARS-CoV-2 3CL^pro^ [23]. Halder et al. found that Saquinavir can target 12 non-structural proteins of SARS-CoV-2, especially key enzymes [24]. Our screening study further revealed that Saquinavir could bind closely to CD13 and PIKfyve, potential targets of SARS-CoV-2. Therefore, Saquinavir could be a potential candidate against COVID-19.

### 3.4 Docking results of Olysio against CD13 and PIKfyve

**Figure 4:**
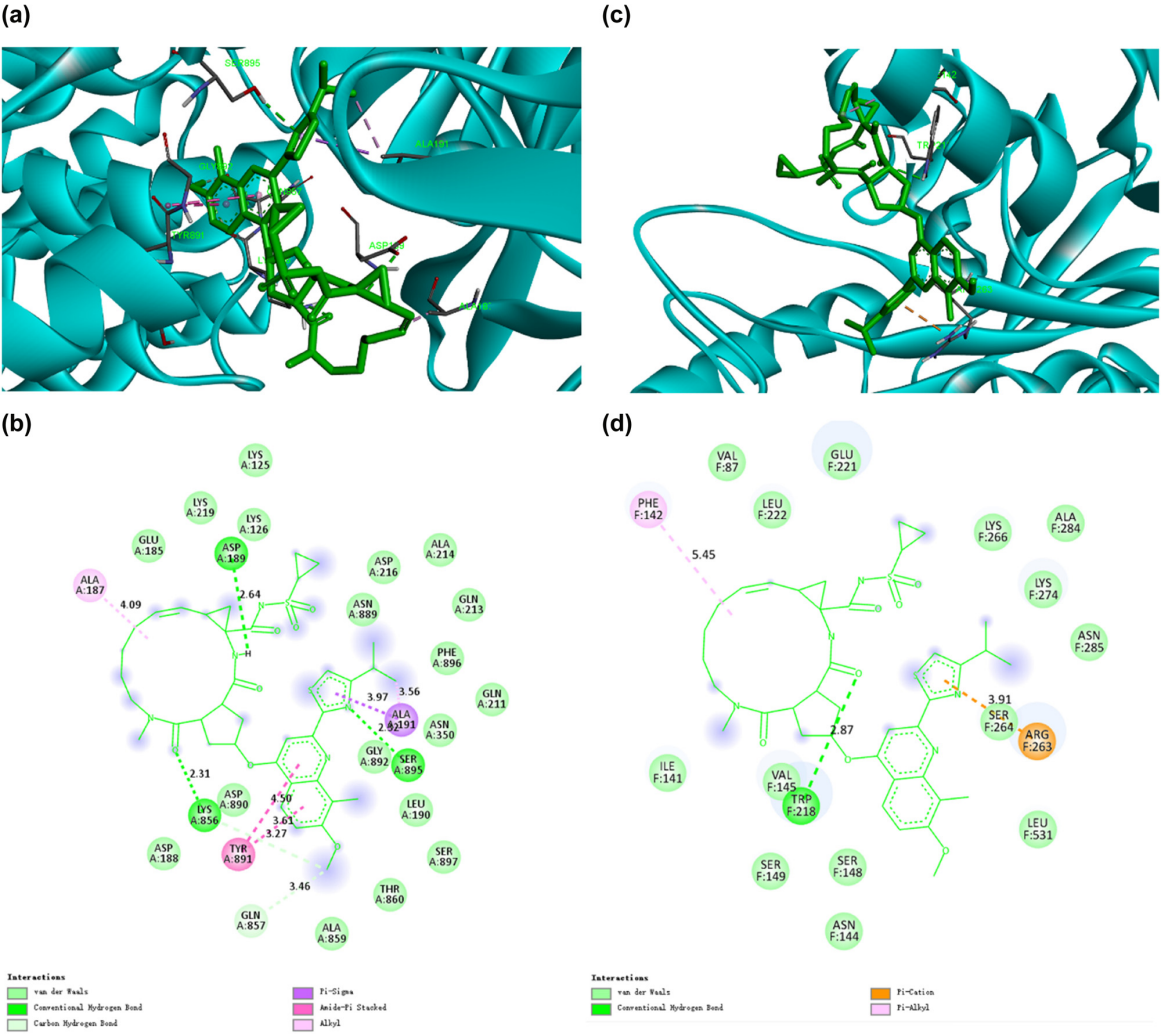
The binding model of Olysio against CD13 and PIKfyve. (a) Interactions between Olysio (green) and associated residues (purple) in the CD13 protein interface pocket (cyan) for SARS‐CoV‐2. (b) Two-dimensional interaction diagram of dihydroergotamine binding to the CD13 active site. (c) Interactions between Olysio (green) and associated residues (purple) in the PIKfyve protein interface pocket (cyan) for SARS‐CoV‐2. (d) Two-dimensional interaction diagram of dihydroergotamine binding to the PIKfyve active site. The numbers next to the dotted line indicate the interaction distance.

Olysio, an HCV inhibitor that targets the NS3/4A protease, is a direct-acting antiviral agent that has been shown to be highly effective in the treatment of HCV infection alone or in combination with other agents [25]. MD simulations have shown that Olysio can bind not only non-structural proteins of SARS but also side chains of residues in the receptor-binding domain binding pocket to inhibit the interaction of viral spike proteins with receptor angiotensin-converting enzyme 2 (ACE2) [26]. In this study, Olysio binding to CD13, a potential SARS‐CoV‐2 target, resulted in 18 van der Waals interactions involving ASP188, ASP890, ALA859, THR860, SER897, LEU190, GLY892, ASN350, GLN211, PHE896, GLN213, ASN889, ASP216, ALA214, LYS126, LYS219, GLU185, and LYS125. It also forms three conventional hydrogen bonds and one carbon–hydrogen bond, involving LYS856, SER895, ASP189, and GLN857. In addition to that, Pi-sigma, amide-Pi stacked, and alkyl are formed with amino acid residues ALA191, TYR891, and ALA187, respectively (Figure 4a and b). For PIKfyve, a potential SARS‐CoV‐2 target, Olysio formed 14 van der Waals interactions when combined with PIKfyve with ILE141, VAL145, SER149, SER148, ASN144, LEU531, SER264, ASN285, LYS274, ALA284, LYS266, GLU221, LEU222, and VAL87. Meanwhile, it made one conventional hydrogen bond with TRP218, one Pi-cation with ARG263, and one Pi-alkyl with PHE142 (Figure 4c and d). Lo et al. found that Olysio could effectively inhibit SARS-CoV-2 replication, reduce SARS-CoV-2 viral load, and synergistically interact with remdesivir in vitro. In addition, cimeprvir not only inhibits major proteolytic enzymes (MPRO) and RNA-dependent RNA polymerase (RdRp) but also modulates host immune responses [27]. The results of this study also indicate that Olysio is a multitarget inhibitor. Therefore, Olysio has great potential as an anti-COVID-19 drug in the future.

### 3.5 Docking results of Raltegravir and Ecteinascidin against CD13

**Figure 5:**
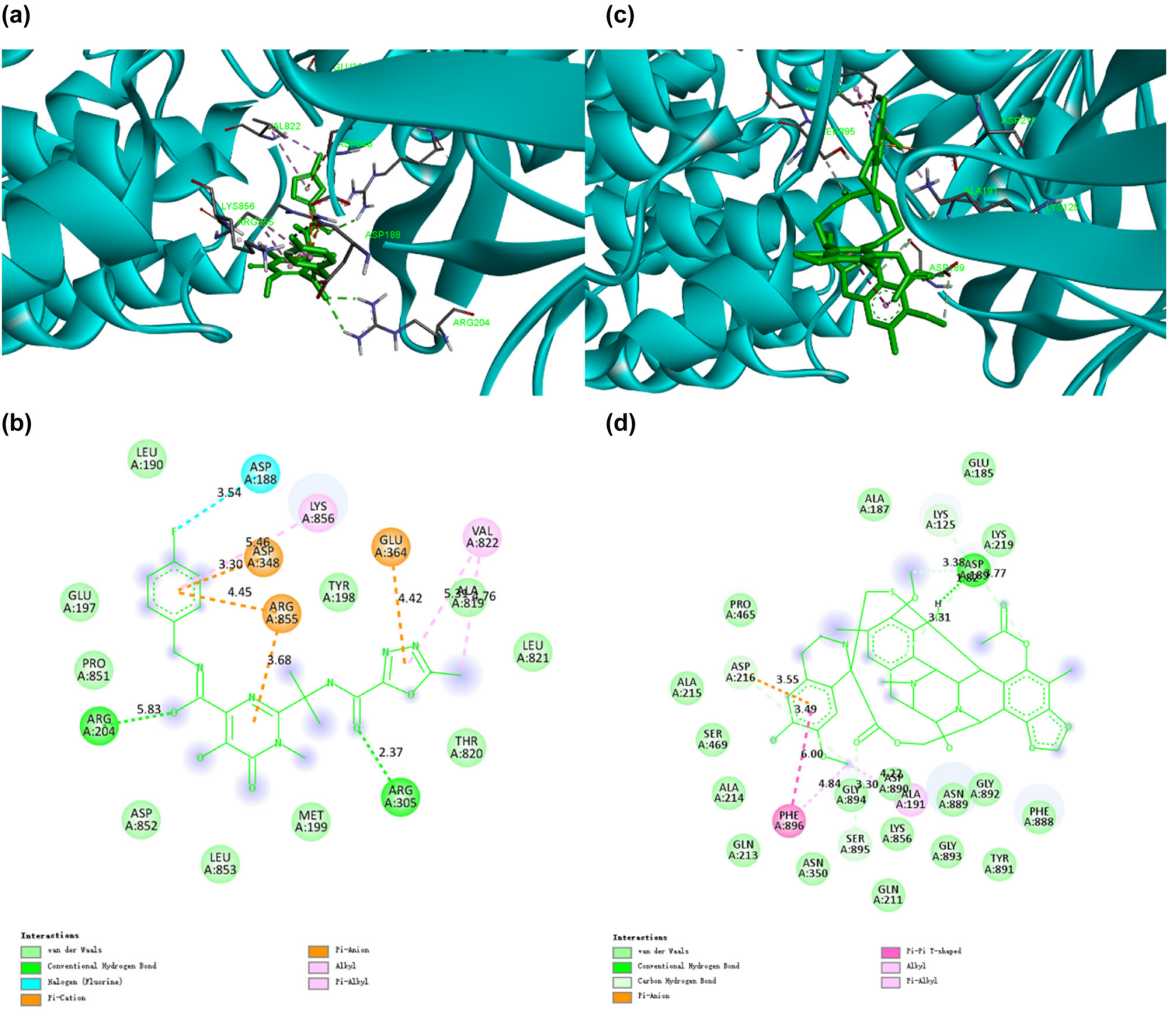
The binding model of Raltegravir and Ecteinascidin against CD13. (a) Interactions between Raltegravir (green) and associated residues (purple) in the CD13 protein interface pocket (cyan) for SARS‐CoV‐2. (b) Two-dimensional interaction diagram of Raltegravir binding to the CD13 active site. (c) Interactions between Ecteinascidin (green) and associated residues (purple) in the CD13 protein interface pocket (cyan) for SARS‐CoV‐2. (d) Two-dimensional interaction diagram of Ecteinascidin binding to the CD13 active site. The numbers next to the dotted line indicate the interaction distance.

Raltegravir was the first integrase chain transfer inhibitor (INSTI), which allows integration into the host cell DNA, approved by the US FDA in 2017 [28]. Akcora-Yildiz et al. showed that Raltegravir is a relatively safe antiviral drug with mild adverse reactions [29]. Ecteinascidin, also known as trabectedin, was discovered in 1969 during screening for antitumor activity in the Caribbean mangrove tunicate *Ecteinascidia turbinate* and was the first marine-derived drug clinically used to treat cancer [30,31]. Our molecular docking studies showed that both Raltegravir and Ecteinascidin could bind to CD13, a potential target of SARS‐CoV‐2, with binding energies of −10.2 kcal/mol, both. When Raltegravir binds to CD13, ten van der Waals interactions are formed, involving amino acid residues LEU190, GLU197, PRO851, ASP852, LEU853, MET199, THR820, LEU821, ALA819, and TYR198. Meanwhile, two conventional hydrogen bonds are formed with ARG204 and ARG305. It also made one Halogen with ASP188. In addition, three cation and anion Pi interactions with amino acid residues ASP348, ARG855, and GLU364 are present. Raltegravir also formed two Pi-alkyl with LYS856 and VAL822 (Figure 5a and b). When Ecteinascidin binds to CD13, 18 van der Waals interactions are formed. What is more, it also formed one conventional hydrogen bond with ASP189 and two carbon hydrogen bonds with SER895 and LYS125. Besides, Ecteinascidin with amino acid residues formed one Pi–Pi t-shaped with PHE896 and one Pi-alkyl with ALA191. Thus, both remdesivir and ascidians bind closely to CD13 (Figure 5c and d). Remdesivir has previously been shown to be effective against SARS-CoV-2 non-structural proteins [32]. Ecteinascidin, a natural Marine drug, showed good binding ability to CD13, suggesting the potential of Marine products as anti-SARS‐CoV‐2 agents. In the future, more ideas for anti-SARS‐CoV‐2 drugs can also be explored along the direction of Marine products.

### 3.6 Docking results of Sitagliptin and Grazoprevir against PIKfyve

**Figure 6:**
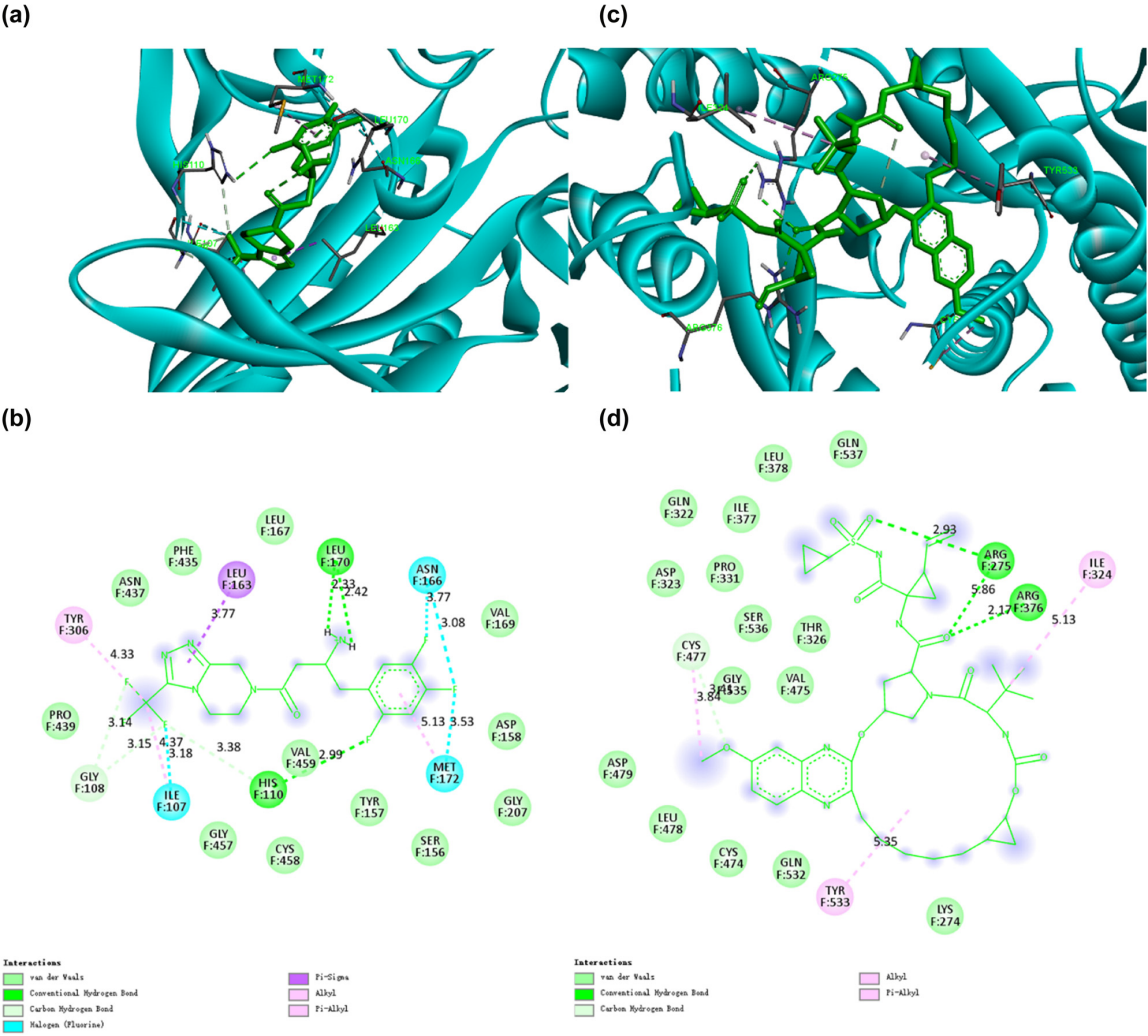
The binding model of Sitagliptin and Grazoprevir against PIKfyve. (a) Interactions between Sitagliptin (green) and associated residues (purple) in the PIKfyve protein interface pocket (cyan) for SARS‐CoV‐2. (b) Two-dimensional interaction diagram of Sitagliptin binding to the PIKfyve active site. (c) Interactions between Grazoprevir (green) and associated residues (purple) in the PIKfyve protein interface pocket (cyan) for SARS‐CoV‐2. (d) Two-dimensional interaction diagram of Grazoprevir binding to the PIKfyve active site. The numbers next to the dotted line indicate the interaction distance.

Sitagliptin, a dipeptidyl peptidase-4 inhibitor, plays an important role in immune regulation, antidiabetes, and anti-inflammation [33,34]. Grazoprevir (MK-5172) is an NS3/4A PI that is highly active against all hepatitis C viruses except genotype 3 [35,36]. Our molecular docking studies showed that both Sitagliptin and Grazoprevir could bind to PIKfyve, a potential target of SARS‐CoV‐2, with binding energies of −9.9 and −9.6 kcal/mol, respectively. When Sitagliptin binds to PIKfyve, 12 van der Waals interactions are formed with amino acid residues. Sitagliptin also made two conventional hydrogen bonds and one carbon–hydrogen bond, involving HIS110, LEU170, and GLY108, respectively. Besides, Sitagliptin and PIKfyve have three Halogen with ILE107, ASN166, and MET172. Meanwhile, one Pi-sigma with LEU163 and one Pi-alkyl with TYR306 are formed (Figure 6a and b). According to Figure 6c and d, when Grazoprevir binds to PIKfyve, 15 van der Waals are formed with amino acid residues. What is more, two conventional hydrogen bonds are formed, involving ARG275 and ARG376. One carbon–hydrogen bond is formed with CYS477. It also formed two Pi-alkyl which involve ILE324 and TYR533. Remarkably, Sitagliptin has been found not only to reduce hepatitis C virus replication in diabetic patients but also to inhibit the production of interferon-induced-protein 10 (CXCL10) chemokine in AIDS patients. However, there is a high level of CXCL10 chemokine in the alveolar microenvironment of patients with COVID-19 [37,38]. Thus, Sitagliptin and Grazoprevir can be used as good potential drugs against SARS‐CoV‐2 and also provide Linchuan researchers with new ideas for drug development.

### 3.7 MD simulation for CD13

**Figure 7:**
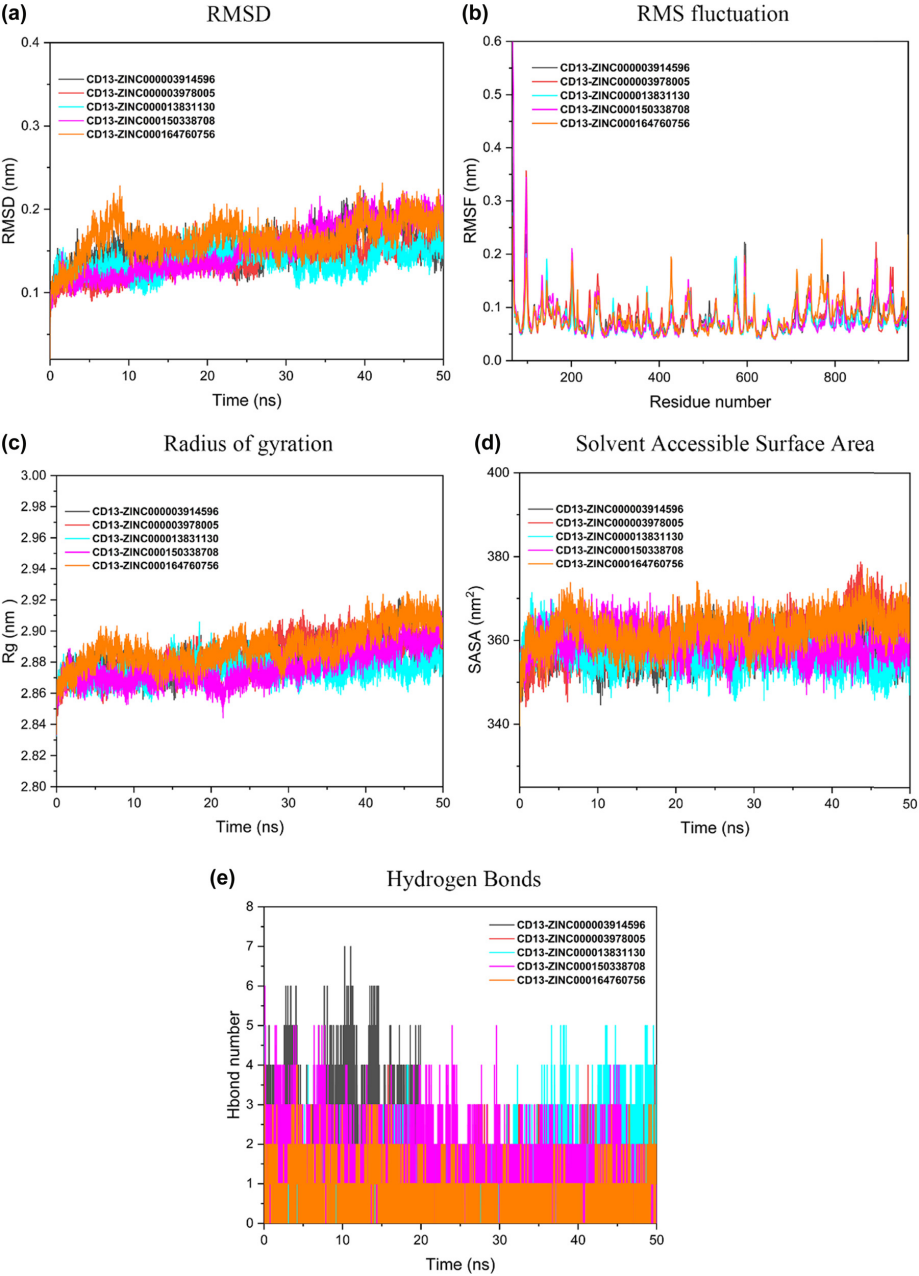
MD trajectory analysis for the selected top five compound–CD13 complexes. (a) RMSD, root mean square deviation. (b) RMSF, root mean square fluctuation. (c) *R*_g_, radius of gyration. (d) SASA, solvent accessible surface area. (e) Hydrogen bonds’ interaction between CD13 and compounds.

We further used MD simulations to investigate the binding stability of protein–ligand docking complexes. We used MD simulations to analyze the docking files between the top five selected compounds and CD13 target proteins to determine the stability and intermolecular interactions between proteins and molecules over a 50-ns time interval. Figure 7a shows the RMSD plots of the five selected CD13 compound complexes. RMSD results showed that the RMSD values of the five complexes ranged from 0.1 to 0.2 nm. The system of the five complexes reached equilibrium after 40 ns, which reflected the good stability of the whole system to a certain extent.

RMSF values for all sampled conformations during the 50 ns simulation were also calculated to determine the degree of ligand deviation from the initial position and protein residue movement. In Figure 7b, the initial residue of the protein (0–100 residues) is not very stable. The final imaging results of the five systems basically overlap between 0.05 and 0.2 nm, and all five CD13 complex complexes have almost similar RMSF patterns. This indicates to some extent that the flexibility of the five systems is low, and the overall effect of composite bundling is better. RMSF results of the five complexes confirmed strong binding to CD13 target proteins.

We evaluated the compactness of the CD13-compound complex by RG analysis. The lower *R*_g_ value indicated that the complex was more compact. We evaluated the compactness of the CD13-compound complex by RG analysis. The lower *R*_g_ value indicated that the complex was more compact. As can be seen from Figure 7c, the five complexes maintained between 2.85 and 2.89 nm at 0–30 ns. However, *R*_g_ values of the five compounds increased slightly after 30 ns. After 45 ns, the *R*_g_ value is basically stable between 2.86 and 2.92 nm. This also shows that the compactness of the protein is constant when the compound binds to the protein and shows a constant interaction during the simulation. *R*_g_ results showed that the selected compounds were tightly bound to CD13 protein.

In Figure 7d, the SASA values of the five complexes varied between 345 and 375 nm^2^. CD13–dihydroergotamine complex and CD13–Olysio complex showed high SASA values compared to other complexes. As we can see from Figure 7e, the formation of hydrogen bonds in the CD13 compound complex shows that hydrogen bond formation is constant when the CD13 compound complex is simulated for 50 ns. Most compounds form more than one hydrogen bond.

### 3.8 MD simulation for PIKfyve

**Figure 8:**
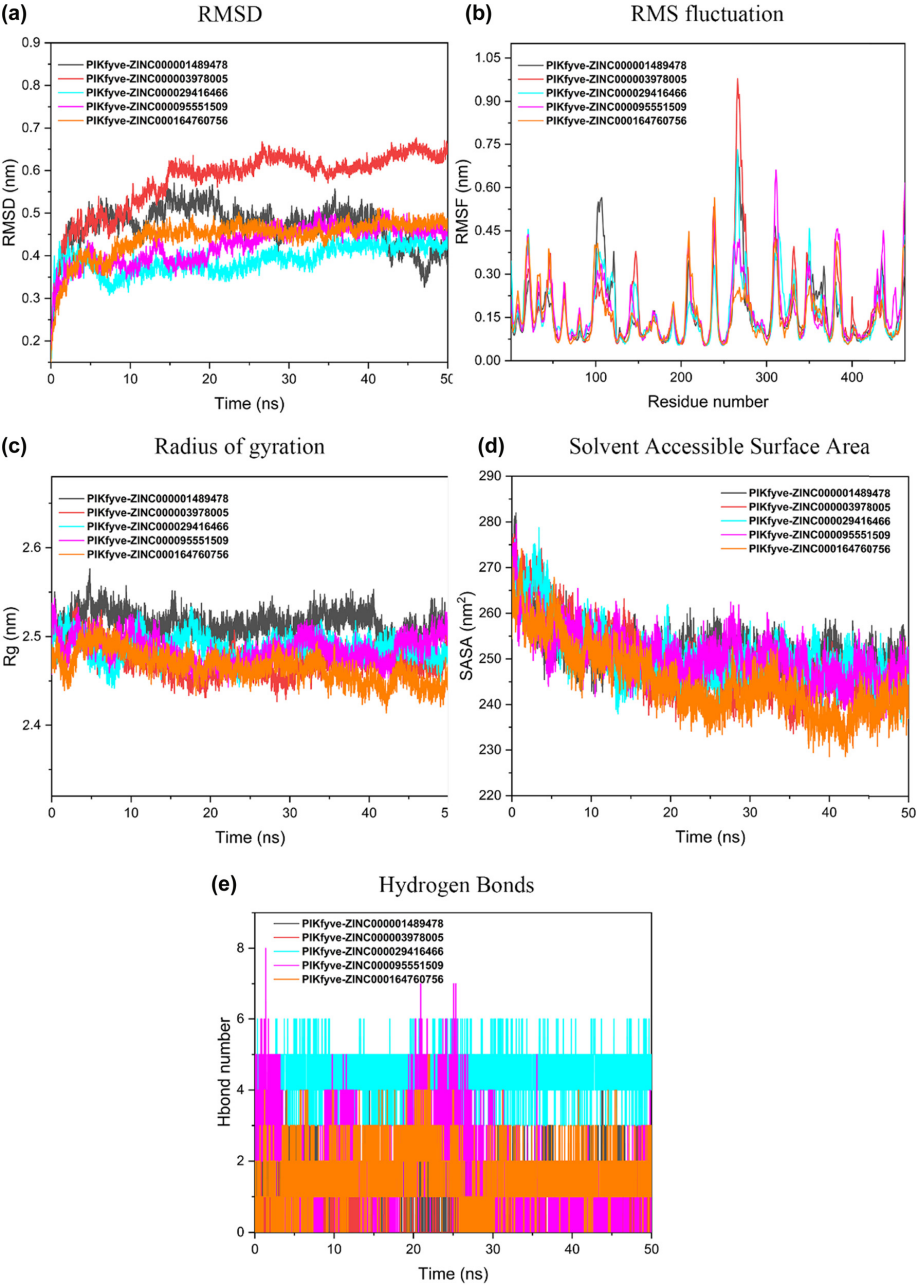
MD trajectory analysis for the selected top five compound–CD13 complexes. (a) RMSD, root mean square deviation. (b) RMSF, root mean square fluctuation. (c) *R*_g_, radius of gyration. (d) SASA, solvent accessible surface area. (e) Hydrogen bonds interaction between CD13 and compounds.

The first five PIKfyve compounds were simulated by RMSD, RMSF, RG, and SASA for 50 ns MD (Figure 8). As shown in Figure 8a, the RMSD value of dihydroergotamine fluctuates greatly from 0 to 15 ns, exceeding 0.3 nm; after 15 ns, the RMSD value is above 0.55 nm, and the overall fluctuation is relatively stable. The other four compounds were also stable after 10 ns. The fluctuation of the root mean square function of five PIKfyve compounds indicates that the compounds have strong binding to the active center of PIKfyve (Figure 8b). All PIKfyve-compound complexes had *R*_g_ values ranging from 2.4 to 2.55 nm. When the compound was bound to PIKfyve, the *R*_g_ values of all complexes showed protein compactness, and all complexes were stable and compact (Figure 8c). In addition, it can be seen from Figure 8d that the reported SASA flat value varies between 230 and 280 nm^2^. After 20 ns, the SASA values of all complexes tended to be stable. Finally, we also calculated the hydrogen bond formation between PIKfyve and the compound. The results showed that all complexes formed more than one hydrogen bond (Figure 8e).

### 3.9 Binding free energy analyses

The binding free energy of seven drugs was calculated by MM/GBSA method through the simulation trajectories of 50 ns MD simulation. The calculated binding free energies of Saquinavir, dihydroergotamine, Raltegravir, Ecteinascidin, and Olysio for the potential target CD13 of SARS‐CoV‐2 were −101.21 ± 11.69, −106.87 ± 13.29, −50.86 ± 18.42, −94.55 ± 15.54, and −84.80 ± 25.66 kcal/mol, respectively, which highlighted dihydroergotamine as the most active one. The van der Waals force was the main driving force for the combination of Saquinavir, dihydroergotamine, Ecteinascidin, and Olysio with CD13 (Table 3). Besides, The calculated binding free energies of Sitagliptin, dihydroergotamine, Saquinavir, Grazoprevir, and Olysio for the potential target PIKfyve of SARS‐CoV‐2 were 93.94 ± 22.86, −107.73 ± 17.65, −184.28 ± 14.22, −119.29 ± 20.82, and −124.35 ± 12.03 kcal/mol. This also indicates that Saquinavir has strong binding activity with PIKfyve. What is more, van der Waals force was the main driving force for the binding of the five compounds to the target protein of PIKfyve (Table 4).

**Table 3:** Calculation results of MM/PBSA of 5 CD13–compound complexes

	Saquinavir	Dihydroergotamine	Raltegravir	Ecteinascidin	Olysio
Δ*E*_vdW_	−205.34 ± 14.53	−192.69 ± 12.79	−184.90 ± 11.61	−191.89 ± 13.01	−254.20 ± 9.71
Δ*E*_ele_	−50.55 ± 8.075	−23.73 ± 12.11	−57.62 ± 12.52	−27.35 ± 8.15	−52.36 ± 13.18
Δ*E*_GB_	179.31 ± 16.65	130.19 ± 14.33	212.58 ± 18.87	145.37 ± 19.67	248.69 ± 33.87
Δ*E*_SA_	−24.64 ± 1.23	−20.62 ± 0.93	−20.92 ± 1.15	−20.68 ± 1.30	−26.93 ± 1.41
Binding energy	−101.21 ± 11.69	−106.87 ± 13.29	−50.86 ± 18.42	−94.55 ± 15.54	−84.80 ± 25.66

*Δ*E*_vdW_ = van der Waals energy terms; Δ*E*_ele_ = electrostatic energy; Δ*G*_GB_ = polar solvation free energy; Δ*G*_SA_ = non-polar contribution to solvation (kcal/mol).

**Table 4:** Calculation results of MM/PBSA of five PIKfyve–compound complexes

	Sitagliptin	Dihydroergotamine	Saquinavir	Grazoprevir	Olysio
Δ*E*_vdW_	−172.20 ± 12.19	−203.74 ± 15.84	−307.57 ± 12.20	−229.80 ± 16.06	−267.46 ± 14.81
Δ*E*_ele_	−58.11 ± 28.35	−17.88 ± 8.00	−95.44 ± 7.72	−16.86 ± 15.56	33.36 ± 16.10
Δ*E*_GB_	146.09 ± 20.13	136.56 ± 11.41	248.91 ± 14.48	153.34 ± 27.14	206.32 ± 38.48
Δ*E*_SA_	−19.73 ± 1.07	−22.66 ± 1.76	−30.18 ± 1.02	−25.97 ± 1.22	−29.86 ± 1.62
Binding energy	−93.94 ± 22.86	−107.73 ± 17.65	−184.28 ± 14.22	−119.29 ± 20.82	−124.35 ± 12.03

## 4 Conclusion

At present, we need to urgently find a specific drug for SARS-CoV-2, so as to ease the inconvenience brought by the novel coronavirus pneumonia to people all over the world. In this study, inhibitors against CD13 and PIKfyve, potential targets of SARS-CoV-2 in the human body, were screened from the FDA-approved compound database through electronic virtual high-throughput screening. Dihydroergotamine, Saquinavir, Olysio, Raltegravir, Ecteinascidin, Sitagliptin, and Grazoprevir have been shown to be candidates for the treatment of SARS-CoV-2 infection. Besides, dihydroergotamine, Saquinavir, and Olysio can simultaneously inhibit CD13 and PIKfyve. Therefore, this study suggested that seven compounds could be further tested for anti-SARS-CoV-2 in vitro, providing more options for the clinical treatment and prevention of COVID-19.
